# Early hepatocellular carcinoma detection using magnetic resonance imaging is cost-effective in high-risk patients with cirrhosis

**DOI:** 10.1016/j.jhepr.2021.100390

**Published:** 2021-11-04

**Authors:** Pierre Nahon, Marie Najean, Richard Layese, Kevin Zarca, Laeticia Blampain Segar, Carole Cagnot, Nathalie Ganne-Carrié, Gisèle N’Kontchou, Stanislas Pol, Cendrine Chaffaut, Fabrice Carrat, Maxime Ronot, Etienne Audureau, Isabelle Durand-Zaleski, ANRS CO12 CirVir, Pierre Nahon, Tarik Asselah, Dominique Guyader, Stanislas Pol, Hélène Fontaine, Georges-Philippe Pageaux, Victor De Lédinghen, Denis Ouzan, Fabien Zoulim, Dominique Roulot, Albert Tran, Jean-Pierre Bronowicki, Thomas Decaensi, Ghassan Riachi, Paul Calès, Jean-Marie Péron, Laurent Alric, Marc Bourlière, Philippe Mathurin, Sebastien Dharancy, Jean-Frédéric Blanc, Armand Abergel, Olivier Chazouillères, Ariane Mallat, Jean-Didier Grangé, Pierre Attali, Louis d’Alteroche, Claire Wartelle, Thông Dao, Dominique Thabut, Christophe Pilette, Christine Silvain, Christos Christidis, Eric Nguyen-Khac, Brigitte Bernard-Chabert, Sophie Hillaire, Vincent Di Martino, ANRS CO22 Hepather, Delphine Bonnet, Virginie Payssan-Sicart, Chloe Pomes, François Bailly, Marjolaine Beaudoin, Dominique Giboz, Kerstin Hartig-Lavie, Marianne Maynard, Eric Billaud, David Boutoille, Morane Cavellec, Marjorie Cheraud-Carpentier, Isabelle Hubert, Jaouad Benhida, Adrien Lannes, Françoise Lunel, Frédéric Oberti, Nathalie Boyer, Nathalie Giuily, Corinne Castelnau, Giovanna Scoazec, Aziza Chibah, Sylvie Keser, Karim Bonardi, Anaïs Vallet-Pichard, Philippe Sogni, Juliette Foucher, Jean-Baptiste Hiriart, Amy Wilson, Sarah Shili, Faiza Chermak, Christelle Ansaldi, Nisserine Ben Amara, Laëtitia Chouquet, Emilie De Luca, Valérie Oules, Rodolphe Anty, Eve Gelsi, Régine Truchi, Elena Luckina, Nadia Messaoudi, Joseph Moussali, Barbara De Dieuleveult, Damien Labarriere, Pascal Poter, Si Nafa Si Ahmed, Véronique Grando-Lemaire, Pierre Nahon, Valérie Bourcier, Séverine Brulé, Thomas Stalhberger, Caroline Jezequel, Audrey Brener, Anne Laligant, Aline Rabot, Isabelle Renard, Thomas F. Baumert, Michel Dofföel, Catherine Mutter, Pauline Simo-Noumbissie, Esma Razi, Hélène Barraud, Mouni Bensenane, Abdelbasset Nani, Sarah Hassani-Nani, Marie-Albertine Bernard, Georges-Philippe Pageaux, Michael Bismuth, Ludovic Caillo, Stéphanie Faure, Marie Pierre Ripault, Christophe Bureau, Jean Marie Peron, Marie Angèle Robic, Léa Tarallo, Marine Faure, Bruno Froissart, Marie-Noelle Hilleret, Jean-Pierre Zarski, Odile Goria, Victorien Grard, Hélène Montialoux, Muriel François, Christian Ouedraogo, Christelle Pauleau, Anne Varault, Tony Andreani, Bénédicte Angoulevant, Azeline Chevance, Lawrence Serfaty, Teresa Antonini, Audrey Coilly, Jean-Charles Duclos Vallée, Mariagrazia Tateo, Corinne Bonny, Chanteranne Brigitte, Géraldine Lamblin, Léon Muti, Abdenour Babouri, Virginie Filipe, Camille Barrault, Laurent Costes, Hervé Hagège, Soraya Merbah, Paul Carrier, Maryline Debette-Gratien, Jérémie Jacques, Guillaume Lassailly, Florent Artu, Valérie Canva, Sébastien Dharancy, Alexandre Louvet, Marianne Latournerie, Marc Bardou, Thomas Mouillot, Yannick Bacq, Didier Barbereau, Charlotte Nicolas, Caroline Chevalier, Isabelle Archambeaud, Sarah Habes, Nisserine Ben Amara, Danièle Botta-Fridlund, Eric Saillard, Marie-Josée Lafrance, Scientific Committee – Voting members, Marc Bourlière, Patrice Cacoub, Fabrice Carrat, Patrizia Carrieri, Elisabeth Delarocque-Astagneau, Victor De Ledinghen, Céline Dorival, Jean Dubuisson, Hélène Fontaine, Chantal Housset, Dominique Larrey, Patrick Marcellin, Philippe Mathurin, Pierre Nahon, Georges-Philippe Pageaux, Jean-Michel Pawlotsky, Ventzislava Petrov-Sanchez, Stanislas Pol, Sophie Vaux, Linda Wittkop, Yazdan Yazdanpanah, Jean-Pierre Zarski, Fabien Zoulim, Jessica Zucman-Rossi, CIRRAL groups, Nathalie Ganne-Carrié, Cendrine Chaffaut, Isabelle Archambeaud, Louis d’Alteroche, Frédéric Oberti, Dominique Roulot, Christophe Moreno, Alexandre Louvet, Thông Dao, Romain Moirand, Odile Goria, Eric Nguyen-Khac, Nicolas Carbonell, Jean-Charles Duclos-Vallée, Stanislas Pol, Victor de Ledinghen, Violaine Ozenne, Jean Henrion, Jean-Marie Péron, Albert Tran, Gabriel Perlemuter, Xavier Amiot, Jean-Pierre Zarski, Sylvie Chevret

**Affiliations:** 1 AP-HP, Hôpitaux Universitaires Paris Seine Saint-Denis, Liver Unit, Bobigny, France; 2 Université Sorbonne Paris Nord, F-93000 Bobigny, France; 3 Inserm, UMR-1138 “Functional Genomics of Solid Tumors”, Centre de recherche des Cordeliers, Université de Paris, Paris, France; 4 Université de Paris, CRESS, INSERM, INRA, URCEco, AP-HP, Hôpital de l’Hôtel Dieu, F-75004, Paris, France; 5 Univ Paris Est Créteil, INSERM, IMRB, Equipe CEpiA (Clinical Epidemiology and Ageing), Unité de Recherche Clinique (URC Mondor), Service de Santé Publique, Assistance Publique Hôpitaux de Paris (AP-HP), Hôpitaux Universitaires Henri Mondor, F-94000, Créteil, France; 6 Clinical Research Department, ANRS | Emerging Infectious Diseases, Paris, France; 7 Université de Paris, département d’hépatologie/Addictologie, Hôpital Cochin, APHP, Paris, France; 8 SBIM, APHP, Hôpital Saint-Louis, Paris, France; 9 Inserm, UMR-1153, ECSTRA Team, Paris, France; 10 Sorbonne Université, Inserm, Institut Pierre Louis d'Epidémiologie et de Santé Publique, AP-HP, Hôpital Saint-Antoine, Unité de Santé Publique, Paris, France; 11 AP-HP, Hôpital Beaujon, Service de Radiologie, Clichy, France

**Keywords:** cirrhosis, MRI, surveillance, liver cancer risk, cost-effectiveness, AFP, alpha-fetoprotein, AMRI, abbreviated magnetic resonance imaging, BCLC, Barcelona Clinic Liver Cancer, HCC, hepatocellular carcinoma, HR, hazard ratio, ICER, incremental cost-effectiveness ratio, LY, life years, LYG, life years gained, MRI, magnetic resonance imaging, NAFLD, non-alcoholic fatty liver disease, QALY, quality-adjusted life year, RFA, radiofrequency ablation, SHR, subdistribution hazard ratio, TACE, transarterial chemoembolization, US, ultrasound

## Abstract

**Background & Aims:**

Reinforced hepatocellular carcinoma (HCC) surveillance using magnetic resonance imaging (MRI) could increase early tumour detection but faces cost-effectiveness issues. In this study, we aimed to evaluate the cost-effectiveness of MRI for the detection of very early HCC (Barcelona Clinic Liver Cancer [BCLC] 0) in patients with an annual HCC risk >3%.

**Methods:**

French patients with compensated cirrhosis included in 4 multicentre prospective cohorts were considered. A scoring system was constructed to identify patients with an annual risk >3%. Using a Markov model, the economic evaluation estimated the costs and life years (LYs) gained with MRI *vs*. ultrasound (US) monitoring over a 20-year period. The incremental cost-effectiveness ratio (ICER) was calculated by dividing the incremental costs by the incremental LYs.

**Results:**

Among 2,513 patients with non-viral causes of cirrhosis (n = 840) and/or cured HCV (n = 1,489)/controlled HBV infection (n = 184), 206 cases of HCC were detected after a 37-month follow-up. When applied to training (n = 1,658) and validation (n = 855) sets, the construction of a scoring system identified 33.4% and 37.5% of patients with an annual HCC risk >3% (3-year C-Indexes 75 and 76, respectively). In patients with a 3% annual risk, the incremental LY gained with MRI was 0.4 for an additional cost of €6,134, resulting in an ICER of €15,447 per LY. Compared to US monitoring, MRI detected 5x more BCLC 0 HCC. The deterministic sensitivity analysis confirmed the impact of HCC incidence. At a willingness to pay of €50,000/LY, MRI screening had a 100% probability of being cost-effective.

**Conclusions:**

In the era of HCV eradication/HBV control, patients with annual HCC risk >3% represent one-third of French patients with cirrhosis. MRI is cost-effective in this population and could favour early HCC detection.

**Lay summary:**

The early identification of hepatocellular carcinoma in patients with cirrhosis is important to improve patient outcomes. Magnetic resonance imaging could increase early tumour detection but is more expensive and less accessible than ultrasound (the standard modality for surveillance). Herein, using a simple score, we identified a subgroup of patients with cirrhosis (accounting for >one-third), who were at increased risk of hepatocellular carcinoma and for whom the increased expense of magnetic resonance imaging would be justified by the potential improvement in outcomes.

## Highlights

• HCC risk stratification in patients with cirrhosis aims at improving performance of cancer surveillance.
• Using a simple scoring system, one-third of patients with cirrhosis with an annual HCC risk >3% can be easily identified.
• Semi-annual surveillance using MRI in this population could enable the cost-effective detection of 5x more BCLC 0 HCCs.

## Graphical abstract

**Figure:**
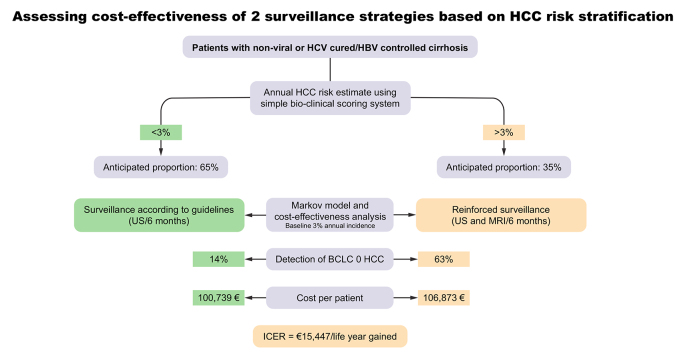

## Introduction

Monitoring for hepatocellular carcinoma (HCC) in patients with cirrhosis based on bi-annual ultrasound (US) examinations^1^ aims to detect liver tumours at the earliest stage possible. This surveillance has been suggested to be cost-effective^2^ and is associated with increased survival.^3^ However, a substantial number of patients included in surveillance programmes are diagnosed with advanced HCC,^4^ particularly because of the poor sensitivity of US (usually less than 30%) in detecting HCC at a very early stage (smaller than 2 cm).^5^ Conversely, it has been shown that more sophisticated techniques, such as magnetic resonance imaging (MRI), can markedly improve the detection of very early-stage HCC^6^; when performed as routine surveillance in patients with cirrhosis, MRI can achieve a detection sensitivity rate of 84.8% for very early-stage HCC compared with 27.3% for US. In this setting, intensification of screening programmes could overcome the pitfalls related to the poor sensitivity of US in patients with cirrhosis. However, implementing surveillance programmes using MRI may only be cost-effective in certain subsets of patients with cirrhosis because of their particularly high annual HCC incidence.^2^ A recent Asian report suggested that using MRI for HCC surveillance was cost-effective in HBV-infected patients with an annual incidence of liver cancer above 3%.^7^ Nevertheless, the results of this study may not be adapted to European populations in which most patients have non-viral causes of liver disease or have been largely exposed to antiviral therapy.

The main driver of the cost-effectiveness ratio is indeed the incidence of HCC in the population considered for screening since it determines the total amount expended on MRI, the receipt of curative treatment, and the overall survival in at-risk patients. Although heterogeneous at the individual level, the changing epidemiology of chronic liver diseases has recently reshaped HCC risk prediction.^8^ This is notably the case in HCV- or HBV-infected patients with cirrhosis, in whom the incidence of HCC has dramatically decreased following the widespread implementation of antiviral agents.^9^^,^^10^ Large prospective cohorts of patients with cirrhosis from Europe and the US have reported global annual HCC incidences ranging from 1.5% to 2.5% following HCV eradication^11^ or HBV control,^9^ which are similar to those observed in patients with non-viral causes of cirrhosis, whether alcohol- or non-alcoholic fatty liver disease (NAFLD)-related.^12^ However, in the era of widespread use of antivirals, the proportion of European patients with a yearly HCC incidence above 3% is currently unknown.

Refining costly HCC screening programmes as a function of HCC incidence is a timely challenge to fairly allocate limited medical resources.^13^ This strategy must however rely on robust data obtained by randomized control trials testing the adjunction of liver MRI for the detection of early-stage HCC. The feasibility of such trials depends on i) the expected proportion of patients with a high HCC incidence in the era of widespread use of antivirals and ii) the cost-effectiveness evaluation of this strategy. Based on data obtained from 4 prospective cohorts of French patients with cirrhosis of various aetiologies included in HCC surveillance programmes,^4^^,^[14], [15], [16] we first estimated the anticipated proportion of patients with a predictable annual liver cancer risk above 3% and then performed an economic evaluation of MRI implementation for early HCC <2 cm detection in this population (*i.e.* Barcelona Clinic Liver Cancer [BCLC] 0 stage) using a previously developed Markov model.^2^

## Patients and methods

### Patients

The present work used data from 1 randomized clinical trial (RCT) dedicated to HCC surveillance and 3 French prospective cohorts of adults with biopsy-proven compensated cirrhosis without detectable suspected focal liver lesions: the CHC2000 trial,^4^ the ANRS CO12 CirVir cohort,^14^ the CIRRAL cohort,^16^ and the ANRS CO22 Hepather cohort.^15^ Each study was conducted in accordance with the ethical guidelines of the 1975 Declaration of Helsinki and French laws for biomedical research and was approved by Ethics Committees. All patients gave written informed consent to participate.

All patients enrolled in these cohorts had periodic liver US surveillance according to international and French guidelines, with or without measurement of alpha-fetoprotein (AFP) serum levels. In the case of detected focal liver lesions, a recalled diagnostic procedure using contrast-enhanced imaging (computed tomography scan or MRI) and/or guided biopsy was performed according to the 2005 AASLD guidelines updated in 2011.^17^^,^^18^ A diagnosis of HCC was thus established by either histological examination or based on probabilistic non-invasive criteria (mainly dynamic imaging revealing early arterial hyperenhancement and washout on portal venous or delayed phases) according to the different time periods (before and after 2011). When HCC diagnosis was established, treatment was determined using a multidisciplinary approach according to AASLD^17^^,^^18^ and the EASL–EORTC^1^ guidelines.

In addition to HCC occurrence, which was the primary endpoint for all 4 cohorts, all events that occurred during follow-up (*i.e.*, death, liver decompensation,^19^ bacterial infection,^20^ extrahepatic malignancies^21^ and cardiovascular diseases^22^) were recorded using information obtained from the medical records of patients held by each centre. Moreover, likely cause(s) of death were established. Patients who underwent liver transplantation were censored for analysis at the date of transplantation. All treatments, including antiviral therapies, were recorded at inclusion, and patients were notified of any modifications during follow-up. A single database encompassing clinical data from the 4 cohorts was built on November 18, 2019.^23^ Among all included patients, only those with non-viral causes of cirrhosis (alcohol-related and/or NAFLD-related) or who achieved HBV control/HCV eradication during follow-up were considered for the present analyses.

#### CHC 2000 trial

The CHC 2000 multicentre randomized study was conducted in 43 tertiary liver centres in France and Belgium to compare 2 US periodicities of surveillance for the detection of small HCC ≤30 mm.^4^ The trial, whose promoter was the Assistance Publique-Hôpitaux de Paris (APHP), was funded by the French Ministry of Health (PHRC 1998 and 2003) and the French Ligue de Recherche contre le Cancer (*ClinicalTrials.gov,* *NCT00190385*). Specific additional inclusion criteria were i) cause of cirrhosis related to either excessive alcohol consumption (80 g per day in males and 60 g per day in females for at least 10 years), chronic infection with HCV (serum anti-HCV antibody-positive), HBV (serum hepatitis B surface antigen-positive), and/or hereditary HFE1 haemochromatosis (C282Y homozygosity) and ii) absence of previous hepatic complications. From June 2000 to March 2006, among the 1,278 randomized and analysed patients, at least 1 focal lesion was detected in 358 patients (28%), but HCC was confirmed in only 123 (9.6%). US surveillance performed every 3 months detected more small focal lesions ≤10 mm than US every 6 months (41% *vs.* 28%, *p* = 0.002) but did not improve the detection of small HCC (*p* = 0.13 between the randomized groups).

#### ANRS CO12 CirVir cohort

The ANRS CO12 CirVir cohort, sponsored and funded by the ANRS (France REcherche Nord & Sud Sida-HIV Hépatites), is a multicentre observational cohort that aims to characterize the incidence of complications occurring in biopsy-proven compensated cirrhosis and to identify the associated risk factors using competing risks analysis.^14^ The full CirVir protocol is available via the ANRS Web site (http://anrs.fr). Specific additional inclusion criteria were i) cause of cirrhosis related to either chronic infection with HCV and/or HBV regardless of the levels of replication and alcohol consumption, ii) patients belonging to Child-Pugh A at enrolment, iii) absence of previous hepatic complications (particularly ascites, gastrointestinal haemorrhage, or HCC), and iv) absence of severe uncontrolled extrahepatic disease resulting in an estimated life expectancy of less than 1 year.

Among 1,822 patients recruited in 35 French clinical centres between March 2006 and July 2012, 151 were subsequently excluded from analysis after reviewing individual data due to either non-compliance with inclusion criteria (n = 142) or consent withdrawal (n = 9), leading to a total of 1,671 patients selected for further analysis.

#### CIRRAL cohort

CIRRAL is a multicentre cohort study implemented in 22 French and 2 Belgian tertiary liver centres to capture the whole spectrum of complications occurring in compensated alcohol-related cirrhosis using competing risk analyses.^16^ The promoter was the APHP. The cohort was funded by the French National Institute of Cancer (INCa), the French Association for Research in Cancer and the ANRS (PAIR CHC 2009) and was registered on ClinicalTrials.gov (NCT00190385). Specific additional inclusion criteria were i) cause of cirrhosis related to chronic alcohol abuse according to the World Health Organization criteria (more than 21 glasses per week for females and more than 28 glasses per week for males) for at least 10 years, ii) absence of chronic infection with HCV or HBV, and iii) patients belonging to Child-Pugh A at enrolment. The follow-up of patients was strictly superposed to the ANRSCO12Cirvir cohort design.

Among 706 patients included between October 2010 and April 2016, 54 were subsequently excluded after reviewing individual data because of violations of the inclusion criteria (n = 48) or consent withdrawal (n = 6); ultimately, a total of 652 patients were selected for further analysis.

#### ANRS CO22 Hepather cohort

The ANRS CO22 Hepather cohort is a French national, multicentre, prospective, observational cohort study of patients with HBV or HCV infection that started in August 2012, among whom 3,045 had active HCV-related cirrhosis at inclusion.^15^ Among the latter, a subset of 1,374 patients consecutively enrolled between 08/2012 and 01/2014 who responded to similar inclusion criteria as those included in the CirVir and CIRRAL cohorts were selected. Follow-up, antiviral treatments, and the definition of the endpoint were identical to those in the CirVir cohort.

### Economic evaluation

**Fig. 1:**
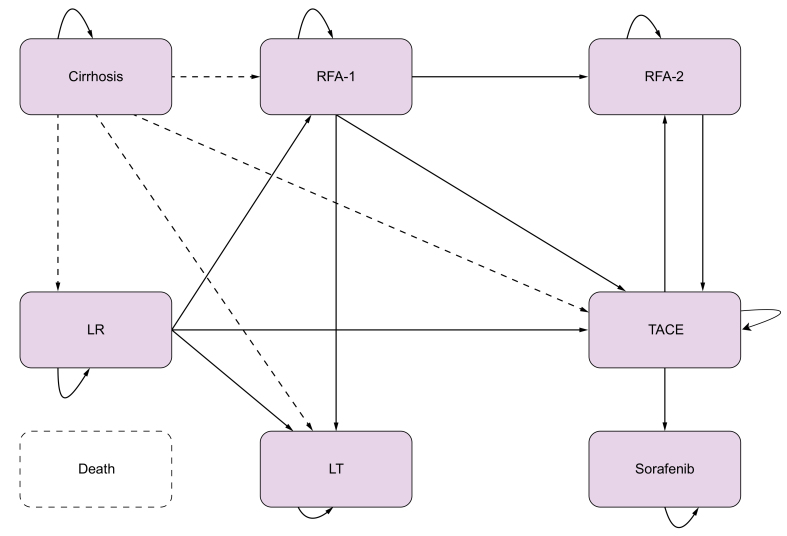
Simplified Markov model. The “posttreatment” states are not represented but are implied in the treatment states for easier graphical representation. LT, liver transplantation; LR, liver resection; RFA, radiofrequency ablation; RFA-1, 1^st^ line RFA; RFA-2, 2^nd^ line RFA; TACE, transarterial chemoembolization. Death can occur in any health state.

We used a previously published Markov model developed to predict the survival of a cohort of 10,000 patients aged 50 years, 65% male, with compensated cirrhosis.^2^ The economic evaluation estimated costs and life years gained with MRI *vs.* US monitoring over a 20-year period and used a 2.5% discount rate for both costs and life years as recommended by the French national health authority (https://www.hassante.fr/upload/docs/application/pdf/202011/methodological_guidance_2020choices_in_methods_for_economic_evaluation.pdf.) The simplified state transition model is presented in Fig. 1 (full model available in Fig. S1) and consists of 14 states: cirrhosis, hepatectomy (tunnel), radiofrequency ablation (RFA, tunnel), RFA for very early stage (tunnel with a count), stable post-RFA, radiofrequency ablation 2 (RFA2) (tunnel), stable post-RFA2, transplantation (tunnel), stable post-transplantation, transarterial chemoembolization (TACE, tunnel), stable post-TACE, sorafenib, and deceased. HCC was not considered a state but as a transition to a treatment depending on disease stage and current practice.

Patients entered the model in the ‘*cirrhosis’* state; the yearly incidence of HCC was set at 3%. Upon being diagnosed with nodules or with liver cancer with either US first followed by MRI or direct MRI, they transitioned to first-line curative-intent treatment for patients in very early and early BLCC stages at diagnosis, using the distribution of treatment presented in Table S1.

Patients who were ineligible for curative-intent first-line treatment received TACE, systemic therapy (sorafenib) or other palliative care. Death was an absorbing state combining disease-specific mortality with age- and sex-specific mortality. The cycle duration was 3 months. Transition probabilities were derived from cumulative probabilities using the declining exponential approximation of life expectancy (DEALE) method. All transition probabilities are presented in Table S2. The sensitivity of US for early HCC detection was established from the ANRS CO12 CirVir cohort. The sensitivity of MRI was drawn from the PRIUS study and was estimated to be 85.7%.^7^

We estimated costs from the viewpoint of the health care system. The cost of managing cirrhosis with <20 mm nodules was estimated on the basis of the annual cost of cirrhosis in the long-term illness scheme of the French Social Health Insurance. In the MRI group, we added the costs of two additional MRIs and specialist consultations each year. The costs of curative HCC treatments were estimated from the French national hospital claims database by extracting admissions corresponding to liver resection, RFA or liver transplantation for which HCC was coded as the primary or secondary diagnosis along with all related readmissions within 3 months of the initial hospitalization. For patients in the stable posttransplant state, the costs of the follow-up were obtained by extracting all hospital stays corresponding to either transplant monitoring or transplant failure 3 months post-transplantation. Other post-transplantation treatments were added.

Palliative treatment combined hospitalization for TACE, systemic therapy (sorafenib) and other palliative care. We identified all hospital stays corresponding to TACE or palliative care for which HCC was coded as the primary or secondary diagnosis as well as readmissions within the next 3 months. As sorafenib is taken at home, we added a specialist consultation to the cost of the drug.

Resource utilization was estimated for each health state and accrued over the 10-year period of the model. Out-of-hospital resources were valued from the SHI tariffs, and hospital stays were the DRG tariff to which daily supplements were added for intensive care. Costs were updated to 2020 values. We calculated the total life years (LYs) accrued by patients and identified the contribution of each strategy to the detection of HCC at very early and early stages. Costs and LYs were discounted at 2.5% yearly. All input values and costs are presented in Tables S2 and S3.

We calculated the incremental cost-effectiveness ratio (ICER) of the MRI surveillance method compared to US. The ICER was calculated by dividing the incremental costs (*i.e*., difference in costs between two treatment options) by incremental LY and explored the uncertainty of the results with deterministic and probabilistic sensitivity analyses. Deterministic analyses were performed on the range of each model parameter; the top 10 most impactful parameters were used to build a tornado diagram. The probabilistic sensitivity analysis was run on all input parameters each using the distribution reported in Table S2.

### Statistical analyses

The baseline was defined as the date of inclusion in the corresponding cohort for patients with non-viral causes of cirrhosis and the date of sustained virological response achievement/viral control for patients with HCV/HBV-related cirrhosis.

Descriptive results are presented as medians (IQR) for continuous variables and as numbers (percentages) for categorical data. The characteristics of patients at the baseline date were compared between the 2 subsets of the cohort using the Student’s *t* test or the Mann-Whitney rank-sum test for continuous variables and the chi-squared test or Fisher’s exact test for categorical variables.

To estimate the proportion of patients with a high risk of HCC set as the first objective of this analysis, a scoring system was developed and validated using the 4 aforementioned cohorts. To this aim, the combined population of the 4 cohorts was randomly divided into a training set (two thirds) and a validation set (one third), using a random sampling with stratification on the initial cohort. The cumulative incidence of HCC was estimated in the training and validation cohorts in a competing risk framework, with death as a competing event.

The multivariate Fine-Gray regression method was used in the training cohort to determine baseline features associated with HCC occurrence, with death as a competing event. Univariate analysis of the influence of features on HCC was performed by computing unadjusted subdistribution hazard ratios (SHRs) along with their 95% CIs using Fine-Gray regression models. The final model was determined in a multivariate analysis by entering all variables associated with HCC risk at the *p* <0.20 level in univariate analysis and then applying a backward stepwise approach to retain significant factors at the *p* <0.05 level. For each variable of this final model, a score was assessed by a linear transformation of the estimated coefficient, after scaling (multiplying) and rounding the coefficients to provide simplified and more useable weights for scoring.

The discrimination performance of the multivariate model was assessed after validation in the validation cohort by computing Wolber's concordance index (C-index), which measures the probability of concordance between predicted and observed survival. Calibration plots were generated to assess the agreement between the observed outcome and the predicted survival probabilities.

Statistical analyses were performed using Stata 16.0 (StataCorp, College Station, TX) and R v4.0.2 (R Foundation for Statistical Computing, Vienna, Austria). *p* values <0.05 were considered statistically significant.

## Results

### Selection and baseline characteristics of patients

A total of 4,973 patients with compensated cirrhosis undergoing HCC surveillance and included in the 4 cohorts were considered (see flow chart, Fig. S2). Among them, 1,302 were excluded, mostly because of persistent HCV/HBV viral infection (n = 1,103) during follow-up. The remaining 3,671 patients had either non-viral causes of cirrhosis and/or cured HCV/controlled HBV infections. Of the latter, 1,158 additional patients were excluded due to missing information in important biological parameters at baseline. Ultimately, 2,513 patients were included in all subsequent analyses (Table 1). The characteristics of the 1,158 excluded patients are displayed in Table S4. The outcomes of the excluded and analysed populations were similar (Fig. S3). The baseline characteristics of the patients are displayed in Table 1 as a function of their inclusion in the training (n = 1,658) or validation (n = 855) set.

**Table 1:** **Baseline characteristics of patients**.

	Training cohort n = 1,658	Validation cohort n = 855	Standardized difference	*p* value
Age (years)	58.5 ± 10.7 58 [51-65.7]	58.7 ± 10.3 58 [52-66]	0.022	0.461
Male sex (n, %)	1,103 (66.5)	587 (68.7)	-0.045	0.281
Platelet count (10^3^/mm^3^)	154.5 [112–202]	150 [109–201]	0.038	0.269
AST (IU)	30 [24-42]	31 [23–43]	-0.021	0.914
AST x N (n = 40)	0.75 [0.60–1.05]	0.78 [0.58–1.08]	-0.021	0.914
ALT (IU)	27 [20-39]	27 [20-40]	-0.002	0.771
ALT x N (n = 40)	0.68 [0.50–0.98]	0.68 [0.50–1.00]	-0.002	0.771
GGT (IU)	54 [30-118]	58 [31-123]	-0.054	0.246
GGT x N (n = 45)	1.20 [0.67–2.62]	1.29 [0.69–2.73]	-0.054	0.246
Prothrombin time (%)	85 [74-95]	84 [74-95]	-0.020	0.936
Albuminemia (g/L)	42 [38.2-45]	42 [39-45]	0.025	0.957
Total bilirubin (μmol/L)	11 [8-17]	11.9 [8-18]	-0.012	0.217
AFP (ng/ml)	5 [3-10.3]	5.1 [3-9.5]	0.037	0.728
Cirrhosis aetiology				0.819
Cured HCV	988 (59.6)	501 (58.6)	0.020	
Controlled HBV	118 (7.1)	66 (7.7)	-0.023	
Alcohol and/or metabolic	552 (33.3)	288 (33.7)	-0.008	
Follow-up (months)	37.0 [IQR: 23.9–58.3]	37.2 [IQR: 23.3–55.6]	0.058	0.488

AFP, alpha-fetoprotein; ALT, alanine aminotransferase; AST, aspartate aminotransferase; GGT, gamma glutamyltransferase.

### HCC incidence and risk factors

After a median follow-up of 37.0 (IQR 23.9–58.3] months in the training set, 131 (7.9%) patients developed HCC (BCLC 0 = 15.9%), with a corresponding yearly incidence of 2.3% (95% CI 1.9–2.7). Similarly, 75 (8.8%, BCLC 0 = 18.2%) HCC cases occurred after 37.2 (IQR 23.3–55.6] months in the validation set (annual incidence = 2.6%; 95% CI 2.1–3.3). The HCC incidence was similar in both sets (SHR = 1.15; 95% CI 0.86–1.52; *p* = 0.347, Fig. S4A). During the same timeframe, 162 (9.8%) patients died in the training set (causes of death: HCC-related 13 [9.4%], liver-related 50 [36.2%], extrahepatic cause 75 [54.3%]; missing data = 24). In the validation set, 83 (9.7%) patients died (causes of death: HCC-related 10 [13.7%], liver-related 26 [35.6%], extrahepatic cause 37 [50.7%]; missing data = 10). Overall survival was also similar in both populations (HR = 1.04; 95% CI 0.80–1.36; *p =* 0.756, Fig. S4B).

Table S5 displays the baseline characteristics of patients as a function of subsequent HCC development and the results from univariate analyses using Fine-Gray regression models. The model selected male sex, older age, lower platelet count, lower prothrombin time, higher bilirubin level, lower albuminemia, and higher aspartate aminotransferase/alanine aminotransferase/gamma glutamyltransferase/AFP levels as HCC risk factors, taking into account competing risks of death. Table S6 shows the thresholds for continuous variables with the highest value of SHR. Table 2 shows the results of multivariate analyses and corresponding SHRs for each variable using Fine-Gray regression models. Male sex, older age, lower platelet count, higher bilirubin level, and higher gamma glutamyltransferase/AFP levels were the final independent HCC predictors selected by the model.

**Table 2:** **HCC risk factors according to multivariate analyses using a Fine-Gray regression model and construction of an HCC risk scoring system (training cohort, n = 1,658)**.

Variables	aSHR [95% CI]	*p* value	Coefficient (ln(SHR))	Score (x3.5)
Age (years)		<0.001		
≤60	Ref.			0
61–65	1.92 [1.21–3.06]	0.006	0.6548859	2
>65	2.96 [2.02–4.32]	<0.001	1.084074	4
Male sex	1.86 [1.23–2.79]	0.003	0.6180754	2
Platelet count ≤120 10^3^/mm^3^	1.81 [1.25–2.62]	0.002	0.5935341	2
GGT>1.5 ULN	2.16 [1.46–3.20]	<0.001	0.7692455	3
Bilirubin >12 μmol/L	1.63 [1.13–2.36]	0.010	0.4897013	2
AFP> 5 ng/ml	2.53 [1.75–3.65]	<0.001	0.9268662	3

AFP, alpha-fetoprotein; GGT, gamma glutamyltransferase; HCC, hepatocellular carcinoma; (a)SHR, (adjusted) subdistribution hazard ratio.

### Estimate of the proportion of high-risk patients with an annual HCC incidence>3%

According to thresholds and SHRs, a corresponding score was applied to each covariate selected by the multivariate model, leading to the calculation of a 16-point scoring system. Table 2 shows the corresponding scoring for each variable. When applied to the training set, the C-indexes for HCC prediction were 79.1 at 1 year, 76.3 at 2 years, 75.0 at 3 years, 75.5 at 4 years, and 73.7 at 5 years. In the validation set, the C-indexes for HCC prediction were 83.1 at 1 year, 74.7 at 2 years, 76.0 at 3 years, 73.8 at 4 years, and 72.3 at 5 years. Fig. S5 shows calibration in both the training and validation sets. The time-dependent sensitivity and specificity of HCC scores in both sets are reported in Tables S7 and S8.

**Fig. 2:**
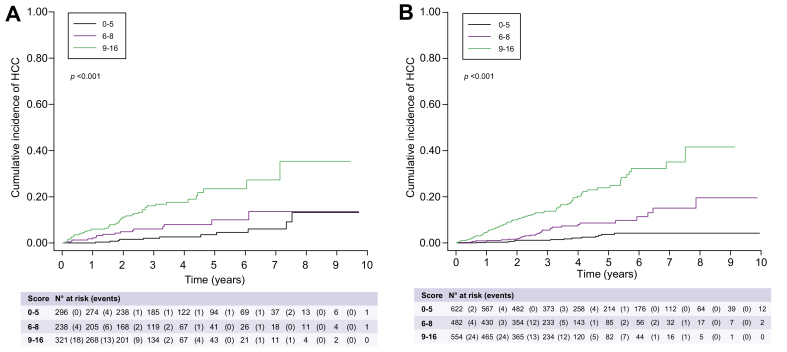
Stratification of HCC risk as a function of the scoring system. (A) In the derivation cohort; (B) In the validation cohort. HCC, hepatocellular carcinoma.

The scores were applied in the training and validation cohorts to identify patients with an annual HCC risk above 3% (Table 3). When applied to the training cohort, patients with a risk score ≥9 had a 5.50% (95% CI 4.45–6.78) annual HCC risk. Below this threshold, the annual HCC incidence was 1.06% (95% CI 0.79– 1.42). The number of patients with a risk score ≥9 was 554, which corresponded to a proportion of patients with an annual HCC incidence above 3% of 33.4% in the training set. Fig. 2A shows the stratification of HCC risk in the development set as a function of the scoring system. In the validation set, application of the same scoring system allowed us to identify 37.5% of patients with a score ≥9 and an annual HCC incidence of 5.36% (95% CI 4.04–7.11) (Table 3). Fig. 2B shows the stratification of HCC risk in the validation set as a function of the scoring system.

**Table 3:** **HCC development as a function of scoring system in the training and validation sets**.

							Cumulative incidence (% [95% CI])				
Score value	No HCC	HCC	Total	sHR	[95% CI]	*p* value	1 year	2 years	3 years	4 years	5 years
Training set (n = 1,658)											
0-5	608 (39.8%)	14 (10.7%)	622 (37.5%)	Ref.			0.3 [0–1.2]	1.1 [0.5–2.3]	1.1 [0.5–2.3]	2.1 [1.0–3.8]	3.7 [2.0–6.1]
6-8	452 (29.6%)	30 (22.9%)	482 (29.1%)	3.38	[1.79; 6.39]	<0.001	0.9 [0.3–2.1]	1.6 [0.7–3.2]	5.4 [3.3–8.1]	7.7 [5.0–11.1]	8.3 [5.4–12.0]
9-16	467 (30.6%)	87 (66.4%)	554 (33.4%)	9.35	[5.30; 16.49]	<0.001	4.6 [3.0–6.7]	9.7 [7.3–12.5]	13.1 [10.2–16.4]	18.8 [14.8–23.3]	21.9 [17.2–27.0]
Validation set (n = 855)											
0-5	285 (36.5%)	11 (14.7%)	296 (34.6%)	Ref			0	1.6 [0.5–3.7]	2.0 [0.8–4.4]	2.6 [1.1–5.3]	3.5 [1.5–6.9]
6-8	222 (28.5%)	16 (21.3%)	238 (27.8%)	2.16	[1.01; 4.60]	0.046	1.8 [0.6–4.2]	4.7 [2.4–8.2]	5.9 [3.2–9.8]	7.7 [4.3–12.5]	9.6 [5.1–15.8]
9-16	273 (35.0%)	48 (64.0%)	321 (37.5%)	5.32	[2.82; 10.03]	<0.001	5.9 [3.7–9.0]	10.9 [7.6–14.9]	15.3 [11.2–20.0]	16.7 [12.2–21.7]	21.6 [15.5–28.5]

HCC, hepatocellular carcinoma.

### Cost-effectiveness analyses

For the baseline incidence of 3% per year, 14% of all cancers detected by US were at a very early stage, *vs.* 63% in the MRI surveillance group (Table 4). For an annual HCC incidence of 3%, patients in the MRI surveillance group gained 13.8 discounted LYs, while those in the US surveillance group gained 13.4 discounted LYs, yielding a difference of 0.4 discounted LYs over the 20-year projection. The total discounted costs per patient were €106,873 and €100,739 for the MRI and US surveillance groups, respectively. The €6,134 difference resulted in an ICER of €15,447/LY.

**Table 4:** **Detection performance of MRI *vs*. US for incidence rates of 3% (baseline), 2% and 1%**. Calculations are based on the Markov simulation over a lifetime horizon for a cohort of 1,000 patients.

Annual incidence of HCC	Strategy	Number of patients diagnosed at BCLC 0 stage	Number of patients diagnosed at BCLC A stage	Number of patients diagnosed at BCLC B, C or D stage	Total HCC
3%	US	59.7	139.8	224.9	424.4
	MRI	268.4	95.3	60.7	424.4
2%	US	43.4	101.5	163.3	308.2
	MRI	194.9	69.2	44.1	308.2
1%	US	23.7	55.4	89.2	168.3
	MRI	106.4	37.8	24.1	168.3

BCLC, Barcelona Clinic Liver Cancer; HCC, hepatocellular carcinoma; MRI, magnetic resonance imaging; US, ultrasound.

**Fig. 3:**
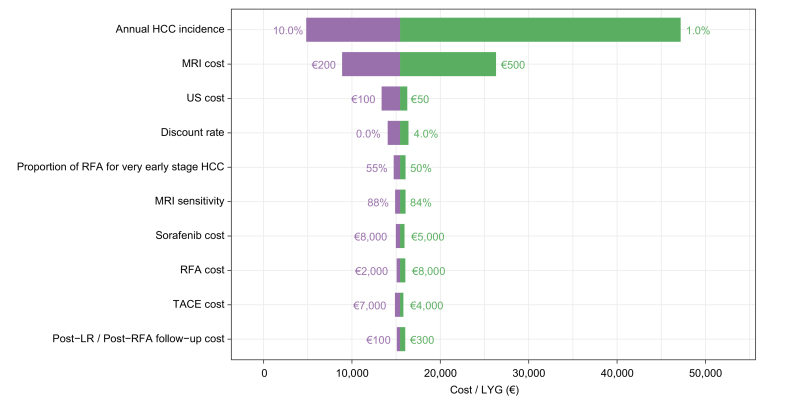
Tornado diagram. The vertical axis represents the point estimate of the incremental cost-effectiveness ratio in €/life year gained. HCC, hepatocellular carcinoma; LR, liver resection; MRI, magnetic resonance imaging; RFA, radiofrequency ablation; TACE, transarterial chemoembolization; US, ultrasound.

**Fig. 4:**
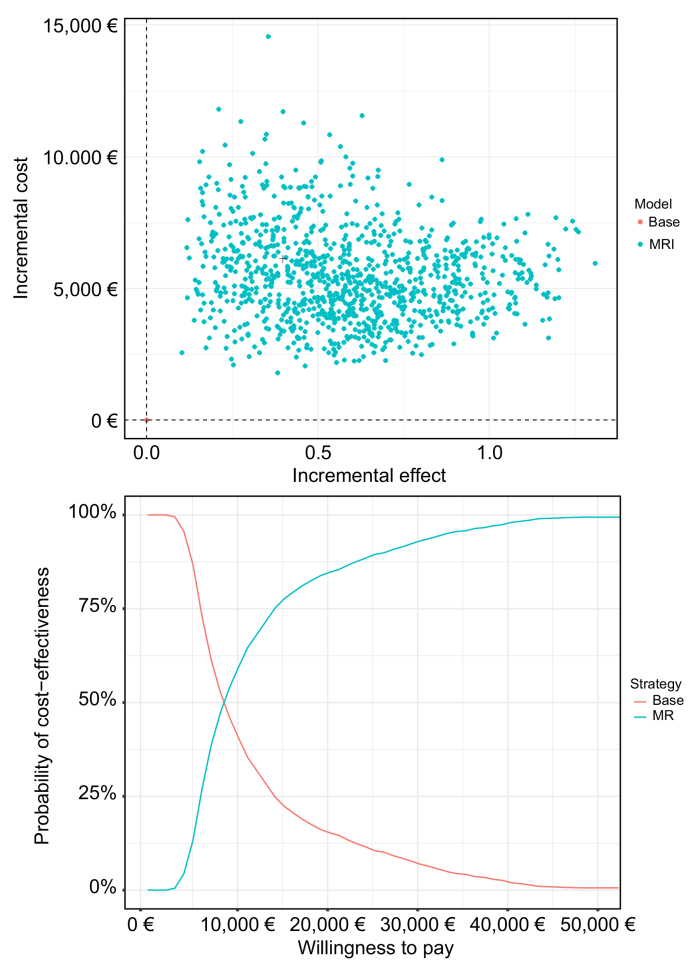
Probabilistic sensitivity analysis and acceptability curves based upon 1,000 replications. The incremental effect is expressed in life years gained, costs are in €, and the incremental cost-effectiveness ratio is expressed in €/LYG. LYG, life years gained; MRI, magnetic resonance imaging.

In the deterministic sensitivity analysis (Table S9), the most influential parameters on the cost-effectiveness of MRI surveillance were the discount rate and the incidence of HCC. For incidence rates of 2% and 1%, the corresponding cost-effectiveness ratios were €23,338/LYG and €47,194/LYG, respectively. The deterministic sensitivity analysis (Fig. 3 and Fig. S6) confirmed the impacts of the annual incidence of HCC and of the unit cost of MRI. Shifting to less expensive technology would further reduce the ICER. The probabilistic sensitivity analysis used 1,000 Monte Carlo simulations. At a willingness to pay of €50,000/LY, MRI screening had a 100% probability of being cost-effective (Fig. 4).

## Discussion

Our prospective cohort- and model-based evaluation of very early HCC detection found that MRI monitoring was cost-effective for a baseline yearly incidence of 3% and over a range of assumptions on incidence, costs and treatment choice. The robustness of the present analyses relies on the study population, which encompassed the following strengths: i) the aggregation of large multicentre cohorts including patients with cirrhosis unambiguously diagnosed using liver biopsy; ii) a prospective design dedicated to HCC surveillance comprising a long follow-up; and iii) the consideration of several therapeutic eras, taking into account the evolution of chronic liver disease management, in particular the widespread use of antiviral therapies. Based on such rigorous aspects, these data enable us to conclude that more than one-third of virus-free patients with cirrhosis are at high risk of HCC development above 3% each year and that early HCC detection using MRI in this subpopulation would be cost-effective. These findings are pivotal for the feasibility of randomized control trials aimed at confirming a potential survival benefit of reinforced surveillance programmes in these patients.

Over the past decades, numerous HCC risk scoring systems aimed at stratifying HBV- or HCV-infected patients into various HCC risk classes have been proposed and validated.^24^ However, most of these risk scores were designed prior to the widespread use of antiviral therapies and are now outdated since they assigned heavy weighting to virological parameters. More recently, new stratification models have been developed in the current era of HCV eradication or HBV control and include routine parameters estimating coexisting comorbidities, persistent liver inflammation or functional impairment.^8^ As described above, the global annual HCC incidence in patients with controlled HBV infection or cured HCV in the case of advanced chronic liver disease is similar to those observed in patients with cirrhosis due to non-viral causes, whether alcohol- or NASH-related.^12^^,^^25^ For instance, using the US Veterans Affairs database,^12^ the annual HCC incidence rates were 1.56% for NAFLD cirrhosis (n = 7,068) and 1.44% for ALD cirrhosis (n = 16,175). Among both cohorts, the HCC risk prediction models incorporated age, sex, diabetes, body mass index, platelet count, serum albumin and transaminases as cancer predictors. These algorithms enabled the stratification of these patients into low (<1%), intermediate (<1%-3%) or high HCC (>3%) yearly risk. Nevertheless, the proportion of patients allocated to each class was not precise. Furthermore, these data were obtained from a registry in which the lack of cirrhosis diagnosis may have introduced selection biases, and details regarding HCC surveillance programmes were not provided.

It is nevertheless tempting to develop universal scoring systems that could be applied regardless of the cause of the underlying liver disease. More recently, an international effort developed a universal HCC risk scoring system using data from 17,374 patients regardless of the cause of the underlying liver disease; the population encompassed HBV- (75%) or HCV-infected patients without viral replication as well as patients with non-viral causes of liver disease who were recruited among 11 international prospective observational cohorts and randomized controlled trials.^26^ The definite algorithm, called the aMAP score, selected older age, male sex, albumin–bilirubin and low platelet count as cancer predictors and was able to identify a high-risk group that accounted for ∼18% of the overall population with an HCC incidence of 19.9% at 5 years. However, this scoring system was not developed in a selected population with cirrhosis. Our analyses conducted in a population of patients with a definite biopsy-proven diagnosis of cirrhosis allowed us to refine this estimate. In addition, the long follow-up and most of the exhaustive records of events (including competing risks of death) in 4 studies dedicated to HCC surveillance strengthen the confidence in the conclusions that were drawn. Finally, the large sample size allowed validation and calibration of the developed models. Overall, our stratification approach reveals that more than one-third of French patients with cirrhosis included in HCC surveillance programmes can be easily identified regardless of the cause of the underlying disease and could be targeted for reinforced screening strategies. Among them, the implementation of MRI as an early detection tool is probably one of the most promising personalized approaches.

MRI is currently only recommended for the characterization of hepatic focal lesions owing to long acquisition times, limited availability and costs. Indeed, although MRI has high sensitivity and specificity for HCC diagnosis, its routine use for surveillance of the cirrhotic population is impaired by health economics issues.^27^ One of the solutions to overcome this pitfall would be to restrict its use to semi-annual surveillance in patients with the highest probability of developing HCC, such as the subpopulation of patients with an HCC yearly incidence above 3%, which is estimated to be one-third of patients with cirrhosis in France. We used a unit cost of 400€ for MRI in the baseline analysis, and the strategy remained cost-effective (below 30,000/LYG) for costs up to €500. Our economic evaluation for French patients confirms the results published by Kim *et al.*^7^ in Asia: MRI allows the detection of HCC at a very early stage and a greater use of treatments with curative-intent. The proportion of curative treatments provided for patients with US detection was higher in our cohort (49% *vs.* 32%) than in Kim *et al.*’s cohort due to our extended use of radiofrequency ablation, which also explains the good ICER, as RFA is the least expensive curative option. This also explains that the difference between US and MRI detection was less favourable in French patients than in Korean patients, with a higher life expectancy for French patients in the US group (13.4 *vs.* 10.7 discounted years). Our sensitivity analyses also indicated that HCC detection with MRI might even be cost-effective in HCC risk below 3%, as suggested by the sensitivity analyses with incidences of 2% and 1% showing values below the accepted threshold of €50,000/LYG. Cost-effectiveness could even be reinforced by the use of abbreviated MRI (AMRI) protocols. Indeed, acquisition of a limited number of sequences for HCC detection with or without contrast-enhanced phases has been shown to be as performant as complete MRI protocols for the early detection of liver cancer.^28^ The anticipated advantages of AMRI include reduced acquisition and interpretation times, which may decrease costs, thus increasing cost-effectiveness if applied for HCC surveillance in the future.

Several limitations must be acknowledged. First, we assumed that patients were adequately monitored in both groups and that the population at risk had full access to MRI. We also applied an average value for sensitivity and did not consider the potential variations in equipment and operator performance. In addition, we assumed the performance of the scoring systems would allow identification of the population at risk and did not include false positives or false negatives of the scoring in our calculations. We did not calculate quality-adjusted life years (QALY) due to the lack of quality-of-life data in the patient population (trial and cohorts). When applying the QALY to life years ratio of the study by Kim *et al.* to our ICER, the incremental cost utility ratio was €26,830 per QALY, close to their $25,202 /QALY for the baseline 3% incidence rate.^7^ Finally, while the consistency with the previous study conducted in Asia supports our findings for the French population, the models rely on hypotheses that are better confirmed by prospective trials and real-life evidence.

In the era of viral suppression or control, the long-term follow-up of patients with cirrhosis included in surveillance programmes has been able to highlight specific subgroups in which HCC risk is sufficiently high to trigger personalized management. This population is estimated to be more than one-third of patients who are usually followed up in tertiary centres and can be easily identified using simple routine measurements, irrespective of the cause of liver disease. MRI using French costs has proven to be cost-effective in this subset of patients and could favour early HCC detection as well as increased allocation to curative procedures. This hypothesis must now be tested in randomized controlled trials dedicated to patients with cirrhosis who are at a high risk of HCC.

## Financial support

The promoters of the 4 prospective cohorts were the 10.13039/501100002738Assistance Publique des Hôpitaux de Paris (APHP) for CHC200 and CIRRAL and 10.13039/501100003323ANRS for CirVir and Hepather. The cohorts were funded by 1) CHC 2000: the French Ministry of Health (PHRC 1998 and 2003) and the French Ligue de Recherche contre le Cancer, 2) the National Agency for Research on HIV and Hepatitis (ANRS) for CirVir and Hepather, and 3) CIRRAL: the 10.13039/501100006364French National Institut of Cancer (INCa), the French Association for Research in Cancer and the 10.13039/501100003323ANRS (PAIR CHC 2009).

## Authors’ contributions

Drs Nahon and Audureau had full access to all data in the study and take responsibility for data integrity and the accuracy of data analysis. *Study concept and design:* Nahon, Audureau, Durand-Zaleski. *Acquisition of data:* Nahon, Layese, Pol, Carrat, Ganne-Carrié, N’Kontchou, Chaffaut. *Analysis and interpretation of data:* Nahon, Layese, Audureau, Durand-Zaleski, Zarca, Najean, Segar. *Drafting of the manuscript:* Nahon, Layese, Audureau, Durand-Zaleski. *Critical revision of the manuscript for important intellectual content:* Nahon, Layese, Cagnot, Ronot, Audureau, Durand-Zaleski. *Statistical analysis:* Layese, Audureau. *Obtained funding:* Dr Jean-Claude Trinchet, Dr Nathalie Ganne-Carrié, Dr Stanislas Pol *Administrative, technical and material support:* Nahon, Cagnot, Layese, Audureau. *Study supervision:* Nahon, Audureau, Durand-Zaleski.

## Data availability statement

The data shown in this article are available from the corresponding authors upon a reasonable request.

## Role of the sponsors

The funding sponsors had no role in the design and conduct of the study; the collection, management, analysis or interpretation of the data; or the preparation, review or approval of the manuscript.

## Acknowledgements

### ANRS CO12 CirVir group∗

Pierre Nahon^1^, Tarik Asselah^2^, Dominique Guyader^3^, Stanislas Pol^4^, Hélène Fontaine^4^, Georges-Philippe Pageaux^5^, Victor De Lédinghen^6^, Denis Ouzan^7^, Fabien Zoulim^8^, Dominique Roulot^9^, Albert Tran^10^, Jean-Pierre Bronowicki^11^, Thomas Decaensi^12^, Ghassan Riachi^13^, Paul Calès^14^, Jean-Marie Péron^15^, Laurent Alric^16^, Marc Bourlière^17^, Philippe Mathurin^18^, Sebastien Dharancy^18^, Jean-Frédéric Blanc^19^, Armand Abergel^20^, Olivier Chazouillères^21^, Ariane Mallat^22^, Jean-Didier Grangé^23^, Pierre Attali^24^, Louis d’Alteroche^25^, Claire Wartelle^26^, Thông Dao^27^, Dominique Thabut^28^, Christophe Pilette^29^, Christine Silvain^30^, Christos Christidis^31^, Eric Nguyen-Khac^32^, Brigitte Bernard-Chabert^33^, Sophie Hillaire^34^, Vincent Di Martino^35^.

^1^AP-HP, Hôpital Avicenne, Service d’Hépatologie, Bobigny, Université Paris 13, Bobigny et INSERM U1162, Université Paris 5, Paris; ^2^AP-HP, Hôpital Beaujon, Service d’Hépatologie, and University Paris Diderot, Sorbonne Paris Cité, CRI, UMR 1149; ^3^CHU Pontchaillou, Service d’Hépatologie, Rennes; ^4^AP-HP, Hôpital Cochin, Département d’Hépatologie et INSERM UMS20 et U1223, Institut Pasteur, Université Paris Descartes, Paris; ^5^Hôpital Saint Eloi, Service d’Hépatologie, Montpellier; ^6^Hôpital Haut-Lévêque, Service d’Hépatologie, Bordeaux; ^7^Institut Arnaud Tzanck, Service d’Hépatologie, St Laurent du Var; ^8^Hôpital Hôtel Dieu, Service d’Hépatologie, Lyon; ^9^AP-HP, Hôpital Avicenne, Service d’Hépatologie, Bobigny; ^10^CHU de Nice, Service d’Hépatologie, et INSERM U1065, Université de Nice-Sophia-Antipolis, Nice; ^11^Hôpital Brabois, Service d’Hépatologie, Vandoeuvre-les-Nancy; ^12^Hôpital Michallon, Service d’Hépatologie, Grenoble; ^13^Hôpital Charles-Nicolle, Service d’Hépatologie, Rouen; ^14^CHU d’Angers, Service d’Hépatologie, Angers; ^15^Hôpital Purpan, Service d’Hépatologie, Toulouse; ^16^CHU Toulouse, Service de Médecine Interne-Pôle Digestif UMR 152, Toulouse; ^17^Hôpital Saint Joseph, Service d’Hépatologie, Marseille; ^18^Hôpital Claude Huriez, Service d’Hépatologie, Lille; ^19^Hôpital St André, Service d’Hépatologie, Bordeaux; ^20^Hôpital Hôtel Dieu, Service d’Hépatologie, Clermont-Ferrand; ^21^AP-HP, Hôpital Saint-Antoine, Service d’Hépatologie, Paris; ^22^AP-HP, Hôpital Henri Mondor, Service d’Hépatologie, Créteil; ^23^AP-HP, Hôpital Tenon, Service d’Hépatologie, Paris; ^24^AP-HP, Hôpital Paul Brousse, Service d’Hépatologie, Villejuif; ^25^Hôpital Trousseau, Unité d’Hépatologie, CHRU de Tours; ^26^Hôpital d’Aix-En-Provence, Service d’Hépatologie, Aix-En-Provence; ^27^Hôpital de la Côte de Nacre, Service d’Hépatologie, Caen; ^28^AP-HP, Groupe Hospitalier de La Pitié-Salpêtrière, Service d’Hépatologie, Paris; ^29^CHU Le Mans, Service d’Hépatologie, Le Mans; ^30^CHU de Poitiers, Service d’Hépatologie, Poitiers; ^31^Institut Mutualiste Montsouris, Service d’Hépatologie, Paris; ^32^Hôpital Amiens Nord, Service d’Hépatologie, Amiens; ^33^Hôpital Robert Debré, Service d’Hépatologie, Reims; ^34^Hôpital Foch, Service d’Hépatologie, Suresnes; ^35^Hôpital Jean Minjoz, Service d’Hépatologie, Besançon. France.

**∗**This study was sponsored by ANRS (France REcherche Nord & sud SIDA-HIV Hépatites: FRENSH). This work is dedicated to the memory of Professor Jean-Claude Trinchet.

### ANRS CO22 Hepather group∗∗

Delphine Bonnet, Virginie Payssan-Sicart, Chloe Pomes (CHU Purpan, Toulouse, France), François Bailly, Marjolaine Beaudoin, Dominique Giboz, Kerstin Hartig-Lavie, Marianne Maynard (Hospices Civils de Lyon, Lyon, France), Eric Billaud, David Boutoille, Morane Cavellec, Marjorie Cheraud-Carpentier (Hôpital Hôtel-Dieu, Nantes, France), Isabelle Hubert, Jaouad Benhida, Adrien Lannes, Françoise Lunel, Frédéric Oberti (CHU Angers, Angers, France), Nathalie Boyer, Nathalie Giuily, Corinne Castelnau, Giovanna Scoazec (Hôpital Beaujon, Clichy, France), Aziza Chibah, Sylvie Keser, Karim Bonardi, Anaïs Vallet-Pichard, Philippe Sogni (Hôpital Cochin, Paris, France), Juliette Foucher, Jean-Baptiste Hiriart, Amy Wilson, Sarah Shili, Faiza Chermak (Hôpital Haut-Lévêque, Pessac, Bordeaux, France), Christelle Ansaldi, Nisserine Ben Amara, Laëtitia Chouquet, Emilie De Luca, Valérie Oules (Hôpital Saint Joseph, Marseille, France), Rodolphe Anty, Eve Gelsi, Régine Truchi (CHU de Nice, Nice, France), Elena Luckina, Nadia Messaoudi, Joseph Moussali (Hôpital de la Pitié Salptétrière, Paris, France), Barbara De Dieuleveult, Damien Labarriere, Pascal Poter, Si Nafa Si Ahmed (CHR La Source, Orléans, France), Véronique Grando-Lemaire, Pierre Nahon, Valérie Bourcier, Séverine Brulé, Thomas Stalhberger (Hôpital Avicenne, Bobigny, France), Caroline Jezequel, Audrey Brener, Anne Laligant, Aline Rabot, Isabelle Renard (CHU Rennes, Rennes, France), Thomas F. Baumert, Michel Dofföel, Catherine Mutter, Pauline Simo-Noumbissie, Esma Razi (Hôpitaux Universitaires de Strasbourg, Strasbourg, France), Hélène Barraud, Mouni Bensenane, Abdelbasset Nani, Sarah Hassani-Nani, Marie-Albertine Bernard (CHU de Nancy, Nancy, France), Georges-Philippe Pageaux, Michael Bismuth, Ludovic Caillo, Stéphanie Faure, Marie Pierre Ripault (Hôpital Saint Eloi, Montpellier, France), Christophe Bureau, Jean Marie Peron, Marie Angèle Robic, Léa Tarallo (CHU Purpan, Toulouse, France), Marine Faure, Bruno Froissart, Marie-Noelle Hilleret, Jean-Pierre Zarski (CHU de Grenoble, Grenoble, France), Odile Goria, Victorien Grard, Hélène Montialoux (CHU Charles Nicolle, Rouen, France), Muriel François, Christian Ouedraogo, Christelle Pauleau, Anne Varault (Hôpital Henri Mondor, Créteil, France), Tony Andreani, Bénédicte Angoulevant, Azeline Chevance, Lawrence Serfaty (Hôpital Saint-Antoine, Paris, France), Teresa Antonini, Audrey Coilly, Jean-Charles Duclos Vallée, Mariagrazia Tateo (Hôpital Paul Brousse, Villejuif, France), Corinne Bonny, Chanteranne Brigitte, Géraldine Lamblin, Léon Muti (Hôpital Estaing, Clermont-Ferrand, France), Abdenour Babouri, Virginie Filipe (Centre Hospitalier Régional, Metz, France), Camille Barrault, Laurent Costes, Hervé Hagège, Soraya Merbah (Centre Hospitalier Intercommunal, Créteil, France), Paul Carrier, Maryline Debette-Gratien, Jérémie Jacques (CHU Limoges, Limoges, France), Guillaume Lassailly, Florent Artu, Valérie Canva, Sébastien Dharancy, Alexandre Louvet (CHRU Claude Huriez, Lille, France), Marianne Latournerie, Marc Bardou, Thomas Mouillot (Dijon University Hospital, Dijon, France), Yannick Bacq, Didier Barbereau, Charlotte Nicolas (CHU Trousseau, 37044 Tours, France), Caroline Chevalier, Isabelle Archambeaud, Sarah Habes (CHU de Nantes, Nantes, France), Nisserine Ben Amara, Danièle Botta-Fridlund, (CHU Timone, Marseille, France), Eric Saillard, Marie-Josée Lafrance, (CHU de Pointe-à-Pitre, Pointe-à-Pitre, Guadeloupe).

### Scientific Committee – Voting members

Marc Bourlière (Hôpital St Joseph, Marseille), Patrice Cacoub (Hôpital Pitié salpêtrière, Paris, France, Fabrice Carrat (Scientific Coordinator, Hôpital Saint-Antoine, Paris, France), Patrizia Carrieri (INSERM U912, Marseille, France), Elisabeth Delarocque-Astagneau (Inserm UMR1181, Paris), Victor De Ledinghen (Hôpital Haut-Lévêque, Pessac, Bordeaux, France), Céline Dorival (UPMC & INSERM U1136, Paris, France), Jean Dubuisson (Inserm U1019, Lille, France), Hélène Fontaine (Hôpital Cochin, Paris, France), Chantal Housset (Inserm UMR-S938 1 IFR65, Paris), Dominique Larrey (Hôpital Saint Eloi, Montpellier, France), Patrick Marcellin (Hôpital Beaujon, Clichy, France), Philippe Mathurin (CHRU Claude Huriez, Lille, France), Pierre Nahon (Hôpital Avicenne, Bobigny, France), Georges-Philippe Pageaux (Hôpital Saint Eloi, Montpellier, France), Jean-Michel Pawlotsky (Hôpital Henri Mondor, Créteil, France), Ventzislava Petrov-Sanchez (ANRS, Paris, France), Stanislas Pol (Principal Investigator, Hôpital Cochin, Paris, France), Sophie Vaux (Agence Nationale de Santé Publique, Saint Maurice, France), Linda Wittkop (ISPED-INSERM U897, Bordeaux, France), Yazdan Yazdanpanah (Hôpital Bichat Claude Bernard, Paris, France), Jean-Pierre Zarski (CHU de Grenoble, Grenoble, France), Fabien Zoulim (Hospices Civils de Lyon, Lyon, France), Jessica Zucman-Rossi (Inserm U674/1162, Paris).

### Scientific Committee – Non-voting members

Marianne L’hennaff (ARCAT-TRT-5-CHV, France), Michèle Sizorn (SOS hépatites, France); one representative of INSERM-ANRS Pharmacovigilance team, Paris, France (Imane Amri, Alpha Diallo), Mélanie Simony, Carole Cagnot (INSERM-ANRS, Paris, France), one member of Inserm Transfert, Paris, France (Alice Bousselet, Mireille Caralp, Jean-Marc Lacombe), and one representative of each pharmaceutical company (MSD, Janssen, Gilead, Abbvie, BMS, Roche).

**∗∗**Financial support provided by INSERM-ANRS (France REcherche Nord&sud Sida-vih Hepatites), ANR (Agence Nationale de la Recherche), DGS (Direction Générale de la Santé) and MSD, Janssen, Gilead, Abbvie, BMS, Roche. **Sponsor:** Imane Amri, Alpha Diallo, Carole Cagnot, Mélanie Simony (INSERM-ANRS, Paris, France), Ventzi Petrov-Sanchez (coordinator). **Methodology and Coordinating Centre:** Loubna Ayour, Jaouad Benhida, Fabrice Carrat (coordinator), Frederic Chau, Céline Dorival, Audrey Gilibert, Isabelle Goderel, Victorien Grard, Warda Hadi, Georges Haour, Godwin Mawuvi, Léa Mba Mintsa, Grégory Pannetier, François Pinot, Muriel Sudres, François Téloulé (Sorbonne Université & INSERM U1136, Paris, France).

### CIRRAL group∗∗∗

Nathalie Ganne-Carrié^1^, Cendrine Chaffaut^2^, Isabelle Archambeaud^3^, Louis d’Alteroche^4^, Frédéric Oberti^5^, Dominique Roulot^6^, Christophe Moreno^7^, Alexandre Louvet^8^, Thông Dao^9^, Romain Moirand^10^, Odile Goria^11^, Eric Nguyen-Khac^12^, Nicolas Carbonell^13^, Jean-Charles Duclos-Vallée^14^, Stanislas Pol^15^, Victor de Ledinghen^16^, Violaine Ozenne^17^, Jean Henrion^18^, Jean-Marie Péron^19^, Albert Tran^20^, Gabriel Perlemuter^21^, Xavier Amiot^22^, Jean-Pierre Zarski^23^, Sylvie Chevret^2^.

^1^AP-HP, Hôpital Avicenne, Service d’Hépatologie, Bobigny, Université Sorbonne Paris Nord, Bobigny et INSERM U1138, Université de Paris; ^2^SBIM, APHP, Hôpital Saint-Louis, Paris, Inserm, UMR-1153, ECSTRA Team, Paris, France; ^3^Liver, CHU, Nantes, France; ^4^Liver Unit, University Hospital, Tours, France; ^5^Liver Unit, University Hospital, Angers, France; ^6^AP-HP, Hôpital Avicenne, Service de Médecine Interne, Bobigny, Université Sorbonne Paris Nord, Bobigny; ^7^Liver unit, CUB Hôpital Erasme, Université Libre de Bruxelles, Belgium, ^8^Liver Unit, University Hospital, Lille, France, ^9^Liver Unit, University Hospital, Caen, France ; ^10^Liver Unit, University Hospital, Rennes, France ; ^11^Liver Unit, University Hospital, Rouen, France ; ^12^Liver Unit, University Hospital, Amiens, France; ^13^Liver Unit, APHP, CHU Saint-Antoine, Paris, France ; ^14^Liver Unit, APHP, CHU Paul Brousse, Villejuif, France; ^15^Université Paris Descartes; APHP, Liver Unit, Hôpital Cochin; INSERM U1223, Institut Pasteur, Paris, France; ^16^Hepatology Unit, University Hospital, CHU Bordeaux, France; ^17^Liver Unit, APHP, CHU Lariboisière, Paris, France; ^18^Liver Unit, University Hospital, Haine Saint-Paul, Belgium, ^19^Liver Unit, Universitary Hospital Purpan, University Paul Sabatier III, Toulouse ; ^20^Institut National de la Santé et de la Recherche Médicale (INSERM), U1065, Team 8, “Hepatic Complications in Obesity”, Nice, F-06204, Cedex 3, France, University Hospital of Nice, Digestive Centre, Nice, F-06202, Cedex 3, France; ^21^Liver Unit, University Hospital, Béclère, APHP, Clamart, France; ^22^Liver Unit, APHP, CHU Tenon, Paris, France; ^23^Clinique d’hépato-gastroentérologie pôle Digidune CHU de Grenoble, France

**∗∗∗**The promoter of the study was the Assistance Publique des Hôpitaux de Paris (APHP), and the cohort was funded by the Institut National du Cancer (INCa).

## Conflict of interest

Pr Nahon has received honoraria from and/or consults for AstraZeneca, Abbvie, Bayer, Bristol-Myers Squibb, Eisai, Gilead, Ipsen, MSD and Roche. He received research grants from AstraZeneca, AbbVie, Bristol-Myers Squibb and Eisai. Pr Ganne-Carrié consults for and/or received personal fees from Abbvie, Bayer, Gilead, Ipsen, and Shionogi, outside the submitted work. Pr Pol has received grants from Gilead, Roche, Abbvie. He consults for BMS, Janssen Cilag, Gilead, Roche, Merck/Schering-Plough, Abbvie, Vivv, Shinogui, Biotest, LFB. Pr Durand-Zaleski consults for AbbVie, Bristol-Myers Squibb, Janssen and MSD. All other authors report no conflict of interest.

Please refer to the accompanying ICMJE disclosure forms for further details.
