# Supplementary material for: Early hepatocellular carcinoma detection using magnetic resonance imaging is cost-effective in high-risk patients with cirrhosis
Source: JHEP Rep. 2021 Nov 4;4(1):100390. doi: 10.1016/j.jhepr.2021.100390 (PMC8683591; doi:10.1016/j.jhepr.2021.100390)
Supplement: Multimedia component 1 [file mmc1.pdf]

Early hepatocellular carcinoma detection using magnetic resonance imaging is cost-effective in high-risk patients with cirrhosis

Pierre Nahon, Marie Najean, Richard Layese, Kevin Zarca, Laeticia Blampain Segar, Carole Cagnot, Nathalie Ganne-Carrié, Gisèle N'Kontchou, Stanislas Pol, Cendrine Chaffaut, Fabrice Carrat, Maxime Ronot, Etienne Audureau, Isabelle Durand-Zaleski for the ANRS CO12 CirVir, ANRS CO22 Hepather, and CIRRAL groups

Table of contents

Fig. S1 2
Fig. S2 3
Fig. S3 4
Fig. S4 5
Fig. S5 6
Fig. S6 7
Table S1 8
Table S2 9
Table S3 12
Table S4 13
Table S5 14
Table S6 15
Table S7 16
Table S8 17
Table S9 18

Fig. S1. Full Markov Model. The "posttreatment" states are not represented but are implied in the treatment states for easier graphic representation. LT: liver transplantation; LR: liver resection; RFA: radiofrequency ablation; RFA-1: 1st line RFA; RFA-2: 2nd line RFA; TACE: transarterial chemoembolization. Death can occur in any health state.

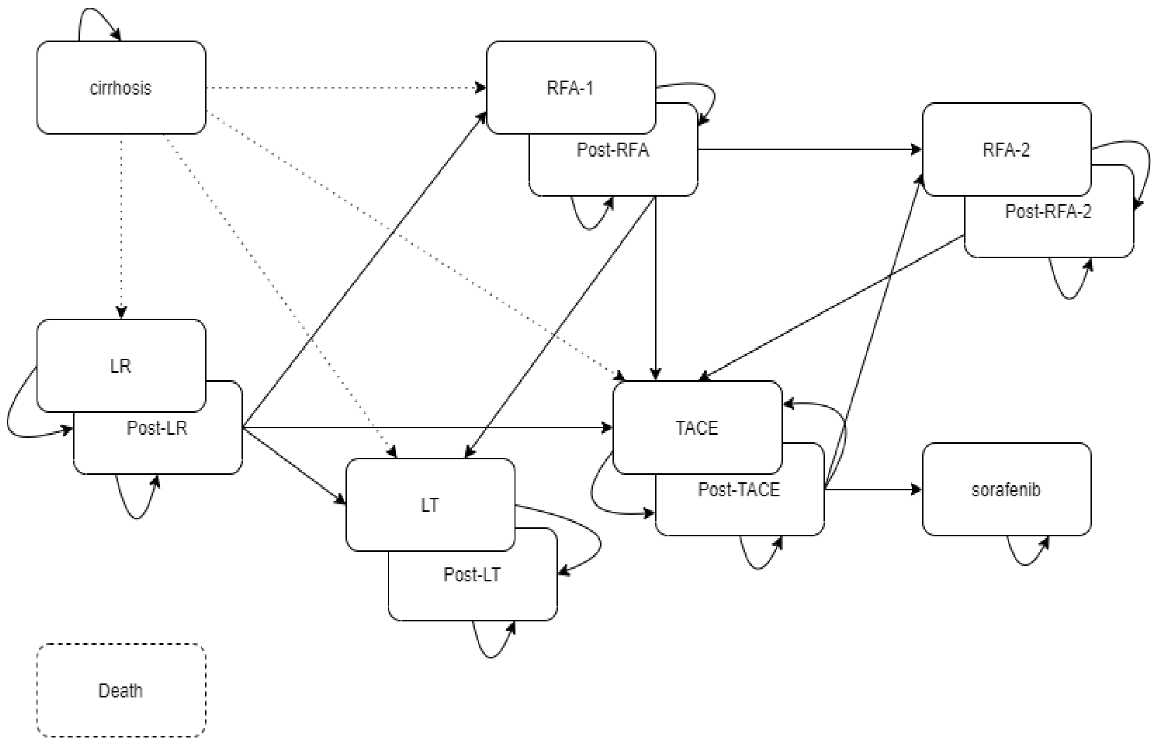

Fig. S2. Flow chart of the study.

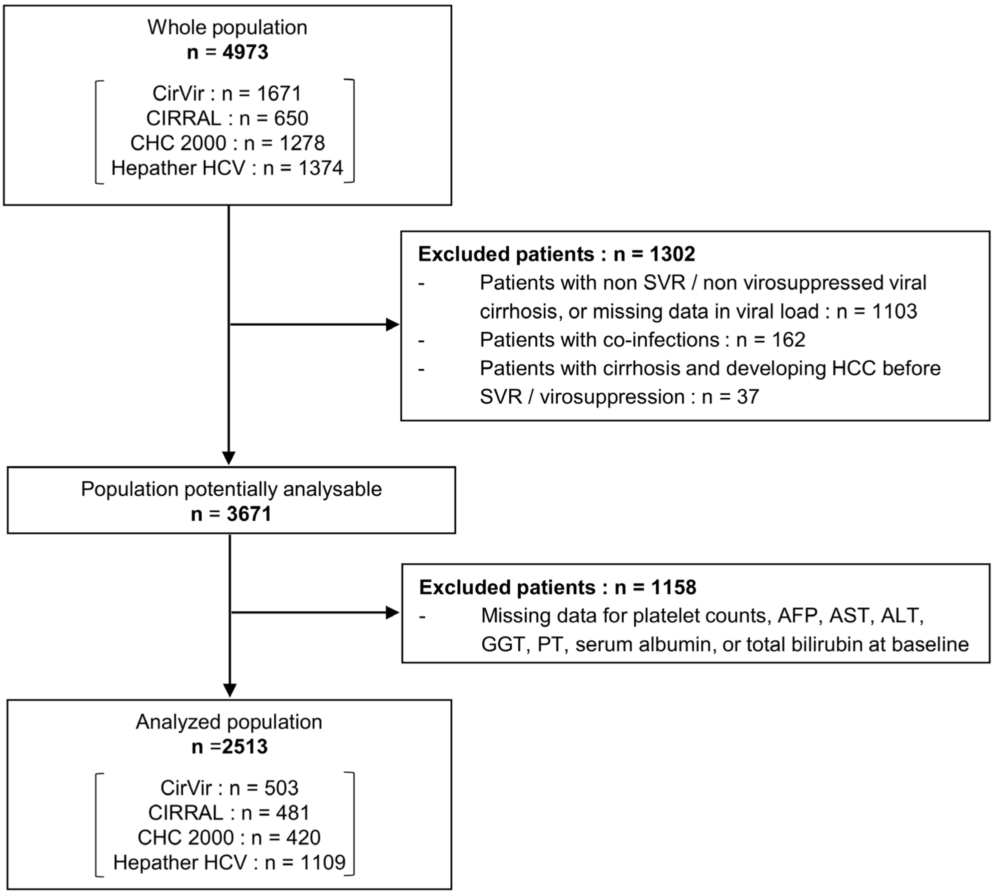

Fig. S3. Outcomes of the excluded and analysed populations: A. HCC incidence; B. Overall survival.

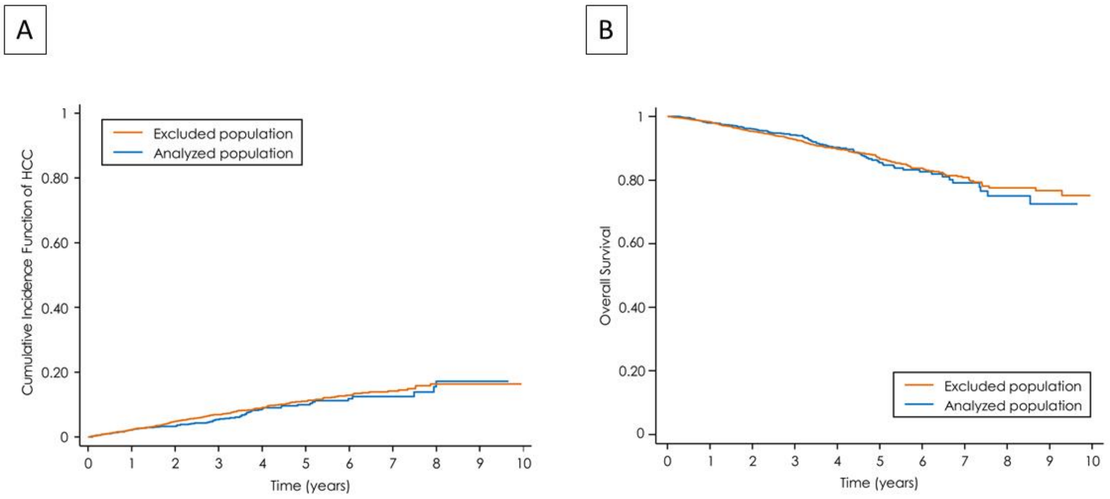

Fig. S4. Outcomes of the training and validation sets: A. HCC incidence; B. overall survival.

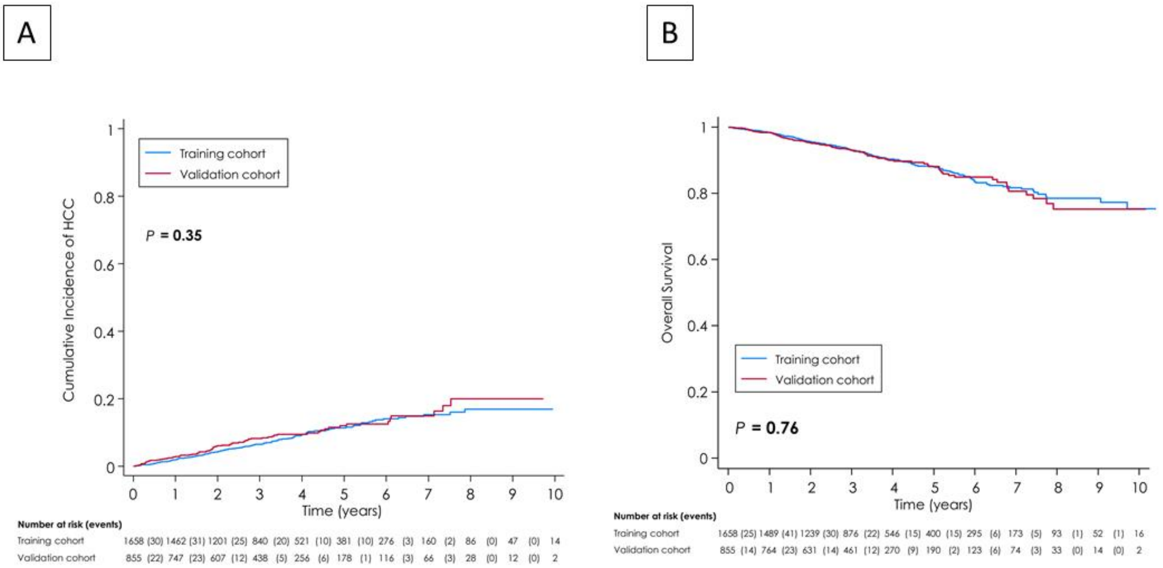

Fig. S5. Calibration of the model in the derivation and validation cohorts.

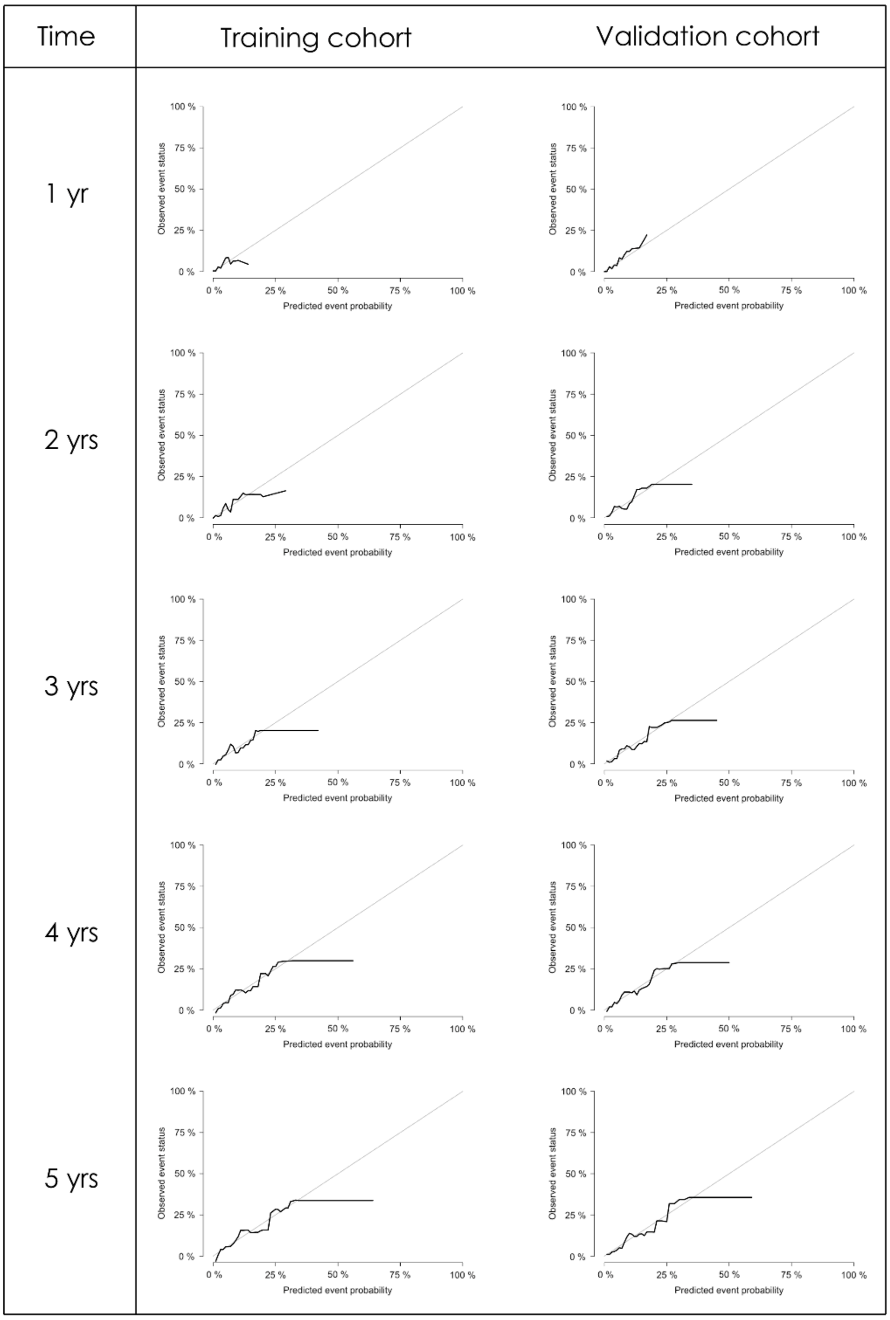

Fig. S6. Full Tornado diagram.

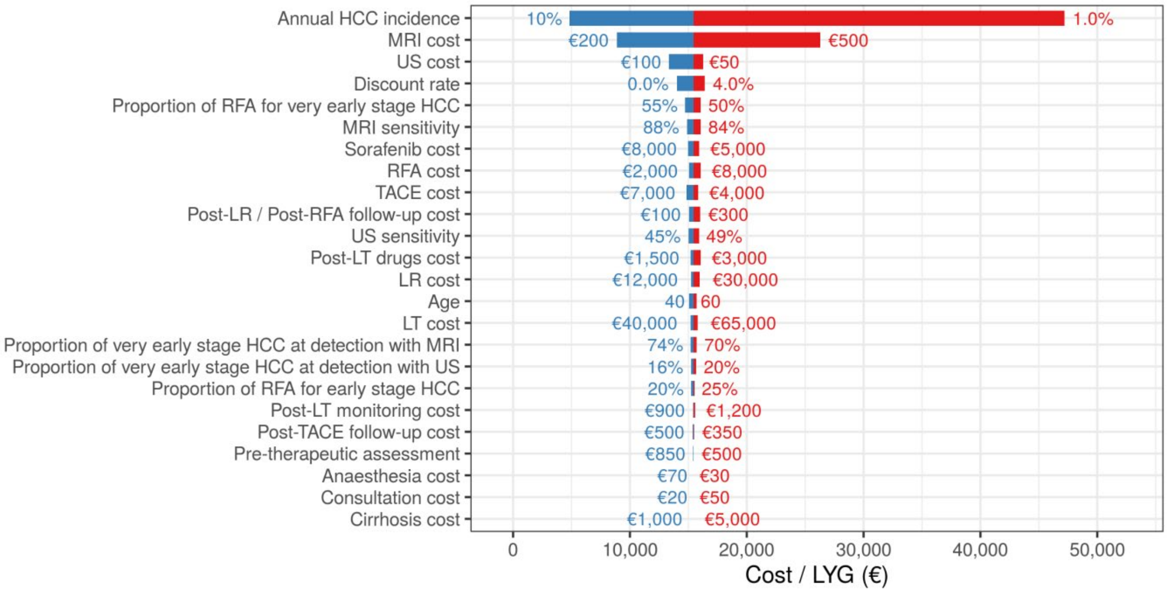

Table S1. Expected distribution of treatments depending on HCC stage at the time of detection.

Treatment	Very early stage (BCLC 0)	Early stage (BCLC A)
Transplantation	4.3%	5.3%
Hepatectomy	13.0%	15.8%
Radiofrequency	52.2%	23.7%
TACE	30.4%	55.3%

Table S2. Transition probabilities and proportions used in the Markov model.

**Markov Model parameters**	**Baseline value**	**Sources**	**One-way sensitivity analysis**	**Probabilistic sensitivity analysis**
Age at model entry (years)	55		40-60	Normal distribution: mean=55, sd=5
Mortality rate	Natural mortality	WHO life tables		
Annual HCC incidence	3%		1-10%	Uniform distribution: min=1, max=10
3-month incidence of HCC	0.7%		*Calculated from annual incidence*	*Calculated from annual incidence*
HCC stages at the time of detection according to screening method (very early = BCLC 0, early = BCLC A)				
MRI				
very early	72.1%	[7]	70%-74%	Uniform distribution: min=70, max=74
early	25.6%	[7]	*Calculated (1 - very early – other)*	*calculated*
others	2.3%	[7]	*2.3%*	*2.3%*
US				
very early	18.5%	[7]	16.5%-20.5%	Uniform distribution: min=16.5, max=20.5
early	43.3%	[7]	*Calculated (1 - very early – other)*	*Calculated*
others	38.2%	[7]	*38.2%*	*38.2%*
MRI sensitivity	85.7%	[7]	83.7%-87.7%	Uniform distribution: min=83.7, max=87.7
US sensitivity	47.0%	[7]	45%-49%	Uniform distribution: min=45, max=49
Treatment proportion in very early-stage HCC				
LT	4.3%	[7]	*4.3%*	*4.3%*
Resection	13.0%	[7]	*13.0%*	*13.0%*
RFA	52.2%	[7]	50%-55%	Uniform distribution: min=50, max=55

TACE	30.4%	[7]	*Calculated*	*Calculated*
Treatment proportion in early-stage HCC				
LT	5.3%	[7]	5.3%	
Resection	15.8%	[7]	15.8%	
RFA	23.7%	[7]	20%-25%	Uniform distribution: min=20, max=25
TACE	55.3%	[7]	*Calculated*	*Calculated*
Treatment proportion in other stages				
TACE	100%	[7]		
Probability of diagnosis with MRI screening				
At very early stage	63.2% †	[7]	†	†
At early stage	22.4% ‡	[7]	‡	‡
At other stages	14.3% §	[7]	§	§
Probability of diagnosis with US screening				
At very early stage	14.0% †	[7]	†	†
At early stage	32.9% ‡	[7]	‡	‡
At other stage	53.0% §	[7]	§	§
Probability of transition for a 3-month cycle				
From cirrhosis with US screening				
To resection	0.7% #	[7]	#	#
To RFA	0.05% *	[7]	*	*
To RFA (very early) *The state "RFA (very early)" is a subset of the state "RFA". It was created to allow counting the patients diagnosed at very early stage in both strategies.*	0.05% **	[7]	**	**
To LT	0.01% ¶	[7]	¶	¶
To TACE	0.9% |	[7]	|	|
To death	Natural mortality	[2]		
From cirrhosis with MRI screening				
To resection	0.08% #	[7]	#	#
To RFA	0.03% *	[7]	*	*
To RFA (very early)	0.2% **	[7]	**	**
To LT	0.02% ¶	[7]	¶	¶
To TACE	0.9% |	[7]	|	|
To death	Natural mortality	[2]		
From resection				
To death	Natural mortality + 0.045	[2]		
From post-resection				
To RFA	0.4%	[2]		
To LT	0.4%	[2]		
To TACE	0.4%	[2]		
To death	Natural mortality	[2]		
From RFA or RFA (very early)				
To death	Natural mortality + 0.012	[2]		
From post-RFA				
To RFA	0.4%	[2]		
To LT	0.4%	[2]		
To TACE	12.7%	[2]		
To death	Natural mortality	[2]		

From RFA-2				
To death	Natural mortality + 0.012	[2]		
From post-RFA-2		[2]		
To LT	1.2%	[2]		
To TACE	0.8%	[2]		
To death	Natural mortality	[2]		
From LT				
To death	8.0%	[2]		
From post-LT				
To death	1.2%	[2]		
From TACE				
To death	5.7%	[2]		
From post-TACE				
To RFA-2	7.3%	[2]		
To TACE	12.5%	[2]		
To sorafenib	6.1%	[2]		
To death	5.7%	[2]		
From sorafenib				
To death	15.9%	[2]		
Discount rate	2.5%		0%-4%	Uniform distribution: min=0, max=4

WHO life tables available at: https://apps.who.int/gho/data/view.main.60580?lang=en

† Sensitivity × [(% of very early stage)/(% of very early stage + % of early stage)].

‡ Sensitivity × [(% of early stage)/(% of very early stage + % of early stage)].

§ 1-Sensitivity.

HCC incidence x [(proba of detection at very early stage x proba of resection at very early stage) + (proba of detection at early stage x proba of resection at early stage)]

* HCC incidence x (proba of detection at early stage x proba of RFA at early stage)

** HCC incidence x (proba of detection at very early stage x proba of RFA at very early stage)

¶ HCC incidence x [(proba of detection at very early stage x proba of transplantation at very early stage) + (proba of detection at early stage x proba of transplantation at early stage)]

| HCC incidence x [(proba of detection at very early stage x proba of TACE at very early stage) + (proba of detection at early stage x proba of TACE at early stage) + (proba of detection at advanced stage x proba of TACE at advanced stage)]

Table S3. Unit costs in 2020 €

Cost (2020 €)			One-way sensitivity analysis	Probabilistic sensitivity analysis (mean; sd)
Cirrhosis	1,855	Social health insurance report 2021 (Yearly ALD cost/4)	1000 - 5000	1,855, 750
Consultation (medical doctor)	28	Social health insurance fee schedule	20-50	28, 5
US	64	Social health insurance fee schedule 2020	50-100	64, 10
MRI	313	Social health insurance fee schedule 2020	200-500	313, 70
Screening programme with US (3-month cycle)	46	(Consultation + US)/2		
Screening programme with MRI (3-month cycle)	170.5	(Consultation + MRI)/2		
Anaesthesiologist consultation	46	Social health insurance fee schedule 2020	30-70	46, 5
Pre-therapeutic assessment	658	[2]	500-850	658, 65
Resection	17,130	[2]	12,000-30,000	17,130, 1,700
Post-resection follow-up	177	[2]	100-300	177, 20
RFA	4,359	[2]	2,000-8,000	4,359, 850
Post-RFA follow-up	177	[2]	100-300	177, 20
LT	51,253	[2]	40,000-65,000	51,253, 5,000
Post-LT follow-up	976	[2]	900-1,200	976, 50
Post-LT drugs	1,953	[2]	1,500-3,000	1,953, 200
TACE	5,172	[2]	4,000-7,000	5,172, 500
Post-TACE follow-up	409	[2]	350-500	409, 40
Sorafenib	6,529	[2]	5,000-8,000	6,529, 600

For the probabilistic sensitivity analysis, gamma distributions were applied to the costs, according to the means and standard deviations presented in this table. The parameters for the gamma distributions were estimated with the method of moments.

Table S4. Comparison of baseline characteristics of analysed versus not analysed populations.

Characteristics	**Available data**	**Analysed population N=2513**	**No analysed population N=1158**	**P value**
Age	3662	58 [51.6-65.9]	57 [51-64.8]	0.006
Age	3662			0.027
≤60		1486 (59.1)	733 (63.8)	
]60 ; 65]		373 (14.8)	154 (13.4)	
>65		654 (26.0)	262 (22.8)	
Male sex	3654	1690 (67.3)	768 (67.3)	0.972
Platelet count	3524	153 [111-201]	155 [106-198]	0.679
Platelet count≤120	3524	777 (30.9)	325 (32.2)	0.477
ASAT	3532	30 [24-42]	32 [24-43]	0.127
ASAT x N (N=40)	3532	675 (26.9)	289 (28.4)	0.364
ALAT	3538	27 [20-39]	27 [20-40]	0.915
ALAT x N (N=40)	3538	580 (23.1)	245 (23.9)	0.600
GGT	3442	56 [31-120]	55 [30-119]	0.988
GGT x 1.5.N (N=45)	3442	1080 (43.0)	387 (41.7)	0.487
TP	3324	85 [74-95]	85 [72-96]	0.757
TP≤90	3324	889 (35.4)	305 (37.6)	0.249
Albumin	3302	42 [38.3-45]	42 [38-44.8]	0.493
Albumin≤40	3302	952 (37.9)	303 (38.4)	0.793
Total bilirubin	3404	11.3 [8-17.1]	12 [8-18]	0.031
Total bilirubin>12	3404	1111 (44.2)	426 (47.8)	0.063
AFP	2880	5.1 [3-10]	4 [2.6-6]	<0.001
AFP > 5	2880	1262 (50.2)	124 (33.8)	<0.001
Cirrhosis aetiology				0.110
Cured HCV		1489 (59.3)	711 (61.4)	
Controlled HBV		184 (7.3)	64 (5.5)	
Alcohol or metabolic		840 (33.4)	383 (33.1)	

Table S5. Baseline characteristics of patients as a function of subsequent HCC development: results from univariate analyses using a Fine-Gray regression models.

Variables	No HCC N=1527	HCC N=131	SHR [95% CI]	P value
Male sex	1002 (65.6)	101 (77.1)	1.69 [1.12 ; 2.54]	0.012
Age (years)	58.2±10.7	62.2±10.1	1.04 [1.02 ; 1.06]	<0.001
Age (years)				<0.001
≤60	925 (60.6)	55 (42.0)	Ref	
]60 ; 65]	228 (14.9)	26 (19.9)	1.88 [1.18 ; 2.98]	0.008
>65	374 (24.5)	50 (38.2)	2.40 [1.64 ; 3.52]	<0.001
Platelet count ($10^3/mm^3$)	156 [115-203]	123 [90-178]	0.993 [0.990 ; 0.996]	<0.001
Platelet count ≤120	436 (28.6)	65 (49.6)	2.47 [1.75; 3.48]	<0.001
AST (UI)	30 [24-40]	37 [28-59]	1.00 [1.00 ; 1.01]	<0.001
AST>40	378 (24.8)	59 (45.0)	2.28 [1.62 ; 3.22]	<0.001
AST>ULN	378 (24.8)	59 (45.0)	2.28 [1.62 ; 3.22]	<0.001
ALT (UI)	27 [20-39]	29 [22-43]	1.01 [1.00 ; 1.01]	0.005
ALT>35	457 (29.9)	53 (40.5)	1.45 [1.02 ; 2.05]	0.036
ALT>0.9 ULN	439 (28.8)	52 (39.7)	1.47 [1.04 ; 2.08]	0.029
ALT>ULN	344 (22.5)	37 (28.2)	1.27 [0.87 ; 1.84]	0.218
GGT (UI)	52 [30-111]	104 [51-181]	1.001 [1.000 ; 1.001]	<0.001
GGT>60	668 (43.8)	92 (70.2)	2.82 [1.93 ; 4.11]	<0.001
GGT>1.5 ULN	613 (40.1)	87 (66.4)	2.77 [1.93 ; 3.99]	<0.001
Prothrombin time (%)	86 [74-95]	78 [70-89]	0.98 [0.98 ; 0.99]	<0.001
Prothrombin time≤90	567 (63.3)	103 (78.6)	2.06 [1.36 ; 3.12]	0.001
Albuminemia (g/L)	42 [38.4-45]	40 [36.3-42.6]	0.96 [0.94 ; 0.97]	<0.001
Albuminemia≤40	568 (37.2)	67 (51.2)	1.79 [1.27 ; 2.52]	0.001
Total bilirubin (µmol/L)	11 [8-16.9]	14 [10-24]	1.01 [1.01 ; 1.02]	<0.001
Total bilirubin >12	629 (41.2)	83 (63.4)	2.34 [1.64 ; 3.34]	<0.001
AFP (ng/mL)	5 [3-10.1]	7 [4-12]	1.00 [0.99 ; 1.00]	0.404
AFP > 5	743 (48.7)	84 (64.1)	2.88 [1.98 ; 4.17]	<0.001

Table S6. Variables and corresponding thresholds used for the association with HCC development (univariate analysis).

**Variables**	**Thresholds**
Age	≤60 and >65
Platelet count	≤120
AFP	>5.
PT	≤90
GGT	>60
GGT ratio	>1.5 ULN
Total bilirubin	>12
AST	>40
AST ratio	>1 ULN
ALT	>35
ALT ratio	>0.9 ULN
Albuminemia	>40

Table S7. Time-dependent sensitivity and specificity of HCC score in the training set.

	1 year		2 years		3 years		4 years		5 years	
Cut-offs	Sens	Spe	Sens	Spe	Sens	Spe	Sens	Spe	Sens	Spe
0	100	3.14	100	3.23	100	3.29	100	4.15	100	5.12
1	100	3.14	100	3.18	100	3.18	100	3.87	100	4.63
2	100	13.01	100	13.34	100	14.43	98.86	16.38	97.75	17.85
3	96.72	16.18	98.48	16.24	99.03	16.42	98.21	17.64	97.20	19.26
4	93.38	25.93	91.81	26.18	94.74	27.64	95.33	30.41	93.41	33.66
5	93.38	37.86	90.05	37.71	93.61	38.96	92.08	39.60	87.87	42.44
6	90.07	45.30	88.52	45.44	88.79	46.82	88.85	47.94	85.17	49.07
7	90.07	59.96	85.09	60.31	78.36	60.33	77.84	60.62	75.99	59.91
8	80.30	67.06	78.98	67.00	70.13	66.78	69.71	67.61	67.69	66.43
9	64.09	78.35	52.64	77.79	48.94	77.17	54.08	78.52	50.94	78.11
10	53.99	84.82	46.22	84.45	44.82	84.32	49.46	85.41	45.33	84.55
11	37.25	90.33	33.50	90.19	32.53	89.92	35.39	90.16	33.60	89.92
12	13.55	96.14	13.75	96.00	15.01	95.66	16.23	96.06	13.53	95.00
13	10.08	97.25	12.15	97.27	13.98	97.27	13.35	97.63	11.13	97.27
14	3.39	99.29	3.52	99.25	5.64	99.19	5.86	99.31	4.88	98.86
15	3.39	99.29	3.52	99.25	5.64	99.19	5.86	99.31	4.88	98.86

Sens: sensitivity, Spe: specificity

Table S8. Time-dependent sensitivity and specificity of HCC score in the validation set.

	1 year		2 years		3 years		4 years		5 years	
Cut-offs	Sens	Spe	Sens	Spe	Sens	Spe	Sens	Spe	Sens	Spe
0	100	3.07	100	3.38	100	3.31	100	3.73	100	3.76
1	100	3.07	100	3.38	100	3.31	100	3.73	100	3.76
2	100	13.11	100	13.98	100	15.14	97.94	17.14	95.91	18.69
3	100	16.73	97.79	17.59	98.38	17.82	96.53	19.09	94.81	20.77
4	100	24.77	91.04	25.83	93.45	27.02	92.23	29.67	91.46	31.59
5	100	35.70	91.04	36.57	91.36	37.26	90.41	37.87	90.05	38.99
6	95.57	43.80	80.03	44.51	83.31	45.80	80.90	45.63	79.28	47.30
7	82.11	57.83	71.76	58.42	77.26	59.76	73.09	57.83	73.20	55.73
8	82.11	63.23	69.60	63.98	71.70	64.33	68.24	63.74	69.43	62.50
9	77.62	74.36	67.56	75.89	65.53	76.50	62.88	72.40	58.06	70.36
10	68.56	82.61	55.92	84.35	55.10	84.45	53.78	81.42	50.98	80.18
11	59.42	86.89	49.40	88.46	48.23	88.63	45.35	86.61	40.16	85.68
12	32.06	94.28	29.72	94.71	31.53	95.96	27.47	94.34	26.24	93.82
13	18.26	96.05	18.62	96.08	18.36	97.00	15.99	96.28	12.45	95.85
14	9.38	99.45	4.26	99.46	3.12	99.49	2.72	99.11	2.11	99.77
15	9.38	99.45	4.26	99.46	3.12	99.49	2.72	99.11	2.11	99.77

Sens: sensitivity, Spe : specificity

Table S9. Deterministic sensitivity analyses (1000 patients)

For a yearly HCC incidence of 1%

**Strategy**	**Cost per patient**	**LYG**	**ICER**
Reference (US)	108,197	14.34	
IRM	115,001	14.48	
ICER			**46,368€/LYG**

For a yearly HCC incidence of 2%

**Strategy**	**Cost per patient**	**LYG**	**ICER**
Reference (US)	103,627	13.84	
IRM	109,898	14.12	
ICER			**22,514€/LYG**
